# Model-agnostic explainable artificial intelligence tools for severity prediction and symptom analysis on Indian COVID-19 data

**DOI:** 10.3389/frai.2023.1272506

**Published:** 2023-12-04

**Authors:** Athira Nambiar, Harikrishnaa S, Sharanprasath S

**Affiliations:** Department of Computational Intelligence, Faculty of Engineering and Technology, SRM Institute of Science and Technology, Kattankulathur, Tamil Nadu, India

**Keywords:** artificial intelligence, machine learning, COVID-19, explainable AI (XAI), data analysis, decision tree, XGBoost, neural network classifier

## Abstract

**Introduction:**

The COVID-19 pandemic had a global impact and created an unprecedented emergency in healthcare and other related frontline sectors. Various Artificial-Intelligence-based models were developed to effectively manage medical resources and identify patients at high risk. However, many of these AI models were limited in their practical high-risk applicability due to their “black-box” nature, i.e., lack of interpretability of the model. To tackle this problem, Explainable Artificial Intelligence (XAI) was introduced, aiming to explore the “black box” behavior of machine learning models and offer definitive and interpretable evidence. XAI provides interpretable analysis in a human-compliant way, thus boosting our confidence in the successful implementation of AI systems in the wild.

**Methods:**

In this regard, this study explores the use of model-agnostic XAI models, such as SHapley Additive exPlanations values (SHAP) and Local Interpretable Model-Agnostic Explanations (LIME), for COVID-19 symptom analysis in Indian patients toward a COVID severity prediction task. Various machine learning models such as Decision Tree Classifier, XGBoost Classifier, and Neural Network Classifier are leveraged to develop Machine Learning models.

**Results and discussion:**

The proposed XAI tools are found to augment the high performance of AI systems with human interpretable evidence and reasoning, as shown through the interpretation of various explainability plots. Our comparative analysis illustrates the significance of XAI tools and their impact within a healthcare context. The study suggests that SHAP and LIME analysis are promising methods for incorporating explainability in model development and can lead to better and more trustworthy ML models in the future.

## 1 Introduction

The world has witnessed the threat of severe acute respiratory syndrome coronavirus disease (COVID-19), which critically affected the public health and economies of many nations over the past 3 years. The pandemic has led to a dramatic loss of human life worldwide and put forth unprecedented challenges to frontline workers. According to the study report by the World Health Organization dated 20 March 2022 (Weekly epidemiological update), over 468 million confirmed cases and over 6 million deaths have been reported globally^1^. Given such an intimidating number of fatalities, it became critical to identify the predictive features and/or risk factors that can aid in early identification of the individuals at risk, thus facilitating the optimal usage of medical resources as well as to deter similar future scenarios.

Motivated by this rationale, various machine learning (ML) based studies as well as exploratory data analysis (EDA) were conducted to analyse the severity of the person, triage measurements, and mortality risk assessment (Barda et al., 2020; Yadav et al., 2020; Ong et al., 2021; Solayman et al., 2023). Although there was limited data access in the early stages due to the confidential nature of data, recently some works have proven their improved potential by incorporating multiple data sources and making it publicly available for research purposes. In particular, various classical machine learning models, i.e., Decision tree, Adaboost, XGboost as well as advanced deep learning models were proposed in various studies (Yadav et al., 2020; Kwekha-Rashid et al., 2021; Solayman et al., 2023) which can predict the future pandemic wave or mortality/recovery of COVID-19 patients of a future pandemic wave.

One of the biggest challenges that exist in many of the aforementioned AI models is the lack of interpretability of the result analysis, which is mainly due to the black-box nature of such models which merely conveys ‘how much' the system performs; not “why” it works. To this end, Explainable Artificial Intelligence (XAI) is proposed to overcome this lacuna by exploring the unexplained hidden “black box” nature of advanced machine learning models and thus providing the reasoning for the model decision. It enables the interpretability of deep-learning-powered models and results in a human-compliant way, hence boosting our confidence in the successful implementation of AI systems in the wild. Such XAI tools can reduce the implementation gap for ML in healthcare, where trust in the model plays a vital part.

In this work, we leverage Explainable Artificial Intelligence (XAI) for COVID-19 data analysis to predict key symptoms that potentially influence on the severity of the disease, which in turn provides insights on medical strategies and opportunities to aid delivery of COVID-19 vaccination priority strategies in the future. In particular, this work presents the interpretable analysis of ML models via model-agnostic XAI models such as SHapley Additive exPlanations (SHAP) values and Local Interpretable Model-Agnostic Explanations (LIME) values, on the COVID-19 symptoms in Indian patients. Three major ML models, i.e., Decision Tree, XGBoost, and Artificial Neural networks are utilized for the development of the classifiers.

Dataset-level performance metrics are calculated for the different ML models to assess the overall performance and the post-hoc explainability tools such as SHAP and LIME are explored further to interpret the results. The main objective of this study is not to identify the most effective model in terms of explainability but rather to shed light on the key considerations to keep in mind while building ML models and to explain the inner workings of these models, not just the output they produce. With this rationale, the proposed XAI models' interpretations are analyzed via various explainability plots. In particular, SHAP-based plots such as global bar plot, local bar plot, beeswarm plot, waterfall plot, and force plot, and LIME-based plots such as local bar plot and violin plot are investigated.

The remainder of the paper is organized as follows: Section 2 details the related works. Section 3 briefly describes the materials and methods that our work is based upon, i.e., dataset description, various AI models, and the XAI methods used in this work. Section 4 details the various experimental studies conducted. Section 5 shows the experimental results including the performance of various AI models and visual analysis of XAI performance. Section 6 and Section 7 explain the state-of-the-art comparisons and discuss significance and future works, respectively. Finally, Section 8 concludes the paper.

## 2 Related work

Explaining the inner workings of deep neural networks has gained significant attention in the past few years. Explainable artificial intelligence (XAI) is an emerging area of research in machine learning that is intended to explore the unexplained hidden “black box” nature of deep neural networks (Guidotti et al., 2018). XAI augments the quality and reliability of model decisions via interpretable evidence, thus shedding light on “why the system works” or “how individual factors contribute to the model's final prediction.”

AI models have played a pivotal role during the COVID-19 pandemic, in exploratory data analysis (EDA), COVID-19 case identification (Adeniyi et al., 2020), identifying the mortality and co-morbidity risks (Snider et al., 2021), prediction of transmission (Lin et al., 2020). Epidemiological models-based studies were conducted in Chen et al. (2020) and Firth et al. (2020) to study and analyze transmission also by relying on many parameters and assumptions. To this end, it became quite important to understand how the individual factors contribute to the final prediction (Casalicchio et al., 2019). Such information is critical in inculcating trust and reliability in the AI model by providing insights into the importance of variables and their relationship with the final prediction.

One major direction of work in XAI includes AI models that are interpretable by design. For instance, ML models such as decision trees (Yan et al., 2020) and logistic regression (Fisman et al., 2020) are used in the studies to identify and interpret the mortality risk prediction of COVID-19 patients, respectively. In the former, a Multi-tree XGBoost model is used to rank the features according to their importance to interpret the model's prediction whereas in the latter a logistic regression model that quantifies the weight of each input variable to the final prediction was realized in order to interpret the importance of the variables. A similar approach based on the logistic regression model was reported in another recent study (Quiroz et al., 2021) leveraging both clinical and imaging data from two hospitals in Hubei, China, for automated severity assessment of COVID-19 for individual patients. That work utilized SHAP values to interpret the co-morbidity conditions and to interpret the severity conditions. Some similar studies are also reported in Petrilli et al. (2020) and Khot et al. (2020). While such “interpretable by design” AI models comprehend the importance of the model's input variables, it was observed that they are often less accurate compared to black-box models (Murdoch et al., 2019; Da Cruz et al., 2021).

Another direction of XAI works focuses on model agnostic interpretation methods that are incorporated into the black-box models to find out the relationship between inputs and the model's prediction. In this regard, some of the major model-agnostic interpretation methods are SHAP (SHapley Additive exPlanations) and LIME (Local Interpretable Model-agnostic Explanations). SHAP uses game-theoretic concepts from economics to assign a Shapley value to each feature, which represents its contribution to the model's prediction and LIME creates a simpler, interpretable model to approximate the original model's behavior, and generates local explanations based on that simpler model. SHAP is designed mainly for tree-based models and neural networks and LIME can be used to interpret any type of machine learning model. One of the works proposed a SHAP-based mortality prediction model upon Israel's COVID-19 patients dataset (Barda et al., 2020). In particular, it investigated the importance of demographic attributes in COVID-19 mortality risk prediction. Another work used the DistilBERT and SHAP approach for COVID-19 infodemic Using Explainable Natural Language Processing (Ayoub et al., 2021). Yet another work by Ong et al. (2021) leveraged image data, i.e., X-ray scans, in order to interpret COVID diagnosis. To this end, they utilized both LIME and SHAP models and compared the results. Similarly, Snider et al. (2021) proposed XGBoost AI model to study COVID-19 instances of patient fatalities in Ontario. In that work, they explored the usage of SHAP value to interpret the model results. A novel image explanation method named Ensemble XAI, a novel image explanation method built on the Grad-CAM++ and SHAP approaches was presented in the work (Zou et al., 2022) for severe community-acquired pneumonia and COVID-19 respiratory infections. A more detailed comparative analysis chart is provided in a later Section 6 and in **Table 5**.

In this work, we leverage model-agnostic XAI tools for interpreting COVID-19 severity prediction in Indian patients from the relevant symptoms. In particular, we investigate the model predictions using three ML approaches, i.e., Decision tree, XGBoost, and Neural Networks. Upon these black-box models, two XAI models—SHAP and LIME—are incorporated to interpret the prediction results. In contrast to similar works on the topic, our work not only presents a more extensive analysis of both global and local XAI models for severity prediction but also facilitates “symptom analysis.” Such a “symptom analysis” helps to comprehend the major symptoms that lead to COVID-19 severity such as tiredness, fever, dry-cough etc. To the best of our knowledge, no other “XAI-for-COVID symptom analysis” work was found in the literature. Furthermore, no similar works using XAI on the COVID dataset was conducted in Indian datasets. Hence, our work marks the first work leveraging XAI tools for COVID-19 data analysis for severity prediction and symptom analysis in the Indian patients' dataset.

## 3 Materials and methods

**Figure 1:**
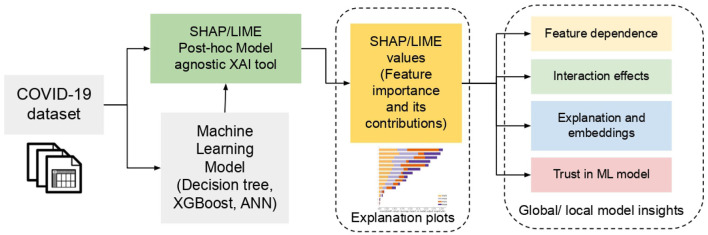
Schematic overview of the Explainable AI framework for COVID-19 severity analysis.

In this section, the datasets and models used in this work are detailed. In particular, data preparation, machine learning models, and the interpretable XAI tools, i.e., SHAP and LIME used to explore the black-box prediction model are explained. The architecture diagram of the proposed Explainable AI framework for COVID -19 severity analysis is depicted in Figure 1. Based on the dataset, first the model is trained for classification. Further, SHAP/LIME- based model-agnostic explainable AI-model is employed upon the classification results, to obtain the interpretation of the results. The detailed overview of each module is presented below:

### 3.1 Dataset description

#### 3.1.1 Data acquisition

Due to the scarcity of literature works reported on the Indian COVID-19 scenario, there was a lack of availability of COVID-19 Indian data for research work. To this end, we carry out extensive web scraping and exploratory data analysis. Various publicly available COVID-19 Indian data from different resources are used to create extensive data regarding COVID-19 patients within India^2^. For instance, data available as open source from IBM^3^ and Kaggle^4^, that contains information such as symptoms of COVID-19, contact with patients, wearing of mask in India etc. during the period 2020–2022. Additionally, we used the data from the work on Exploratory Data Analysis of COVID-19 in India (Mittal, 2020). Various datasets collected from different sources such as Ministry of Health and family Welfare (MOH, 2021), COVID-19 India website (COVID-19 India, 2020), Datameet Data repository (Datameet, 2020), Worldometer (Worldometers, 2020), and WHO (WHO, 2020) are leveraged in that work to study spread and trend of the COVID-19 in India. Among the various data repositories available in that work (Mittal, 2020), we use “Symptoms observed for COVID-19 in India” data. It contains information about the presence of symptoms observed in COVID-19 patients from India.

All data are collected from patient health records as of during the period 1st January 2020 to 1st January 2022. One key assumption we postulate is that the consolidated dataset makes a sample representative database of Indian patients, which are collected from various available Govt./private resources, including data from all Indian states and all genders. Among the total 3,16,000 cases included in the dataset, 2,67,600 patients had severe COVID-19, and the remaining 48,400 either recovered from COVID-19 or remained hospitalized. Each of 22 binary data features is collected in this work. Since different symptoms appear to be independent factors but the severity appears to be a dependent variable in our scenario, we chose severity as the target variable. Of these features, preprocessing is carried out to remove unwanted and/or repetitive data contents, e.g., “Severity_mild,” “Severity_moderate,” “Severity_high,” and “Country” are removed. Once the data is cleaned, the target variable is set to be “Severity_present.” It shows the presence of COVID-19 severity in the patient (either mild, moderate, or high). Hence, “Severity_present” is selected as the target variable, since this variable can help us decide whether the patient is severe or not by using binary classification. Refer to Table 1 for the complete list of 17 input features^5^.

**Table 1:** Summary of the features used in the AI models for the COVID-19 analysis.

**S.No**.	**Attribute/Symptom**
1.	Fever
2.	Tiredness
3.	Dry-Cough
4.	Difficulty-in-Breathing
5.	Sore-Throat
6.	Pains
7.	Nasal-Congestion
8.	Runny-Nose
9.	Diarrhea
10.	None_Symptoms
11.	None_Experiencing
12.	Gender_Female
13.	Gender_Male
14.	Gender_Transgender
15.	Contact_Dont-Know
16.	Contact_No
17.	Contact_Yes

#### 3.1.2 Data cleaning and analysis

Data cleaning is also referred to as data cleansing and data scrubbing. It is one of the most important steps in data processing and decision-making since the quality of the input data has a significant impact on the output. Data cleaning rectifies corrupted/incorrectly formatted, duplicate, or incomplete data in a dataset. It identifies and addresses inaccurate records, improving the dataset by replacing, modifying, or deleting problematic information. The cleaned data is further fed toward the machine learning phase.

### 3.2 AI models

In this work, we leverage three machine learning models to analyse the severity of the disease: (i) Decision tree (Rochmawati et al., 2020); (ii) Extreme gradient boosting decision tree—XGBoost machine learning model (Jiang et al., 2021); and (iii) Artificial Neural Network (Venables et al., 2002). The first two are the classical ML models whereas the latter one is a deep-learning based Neural network model. These models are selected due to their high accuracy performance in binary classification problems and acceptance in many of the previous literature.

**a) Decision tree model**

Decision tree is one of the popular and most widely used algorithms to make predictions based on the input feature data (Huyut and Üstündağ, 2022). The program creates decision trees that describe a set of decisions and their potential outcomes using mathematical formulas like entropy, information gain, and Gini index. The formula for calculating the entropy is:


(1) $$\begin{array}{l} Entropy\left(S\right)=-\overset{n}{\underset{i=1}{\sum }}p_{i}log_{2}p_{i} \end{array}$$

where *S* is the set of examples, *n* represents the number of possible classes or categories that the examples can belong to and *p*_*i*_ is the percentage of examples in class *i*. The above equation is used to compute entropy, which is a measure of the impurity of a set of examples. Similarly, Information Gain (*IG*) is calculated using the formula:


(2) $$\begin{array}{l} IG=Entropy\left(S\right)-\underset{v\in Values\left(A\right)}{\sum }\frac{\left|S_{v}\right|}{\left|S\right|}Entropy\left(S_{v}\right) \end{array}$$

where *S* is the set of examples and *S*_*v*_ is the subset of *S* for which attribute *A* has value *v*. Information Gain(S, A) measures the reduction in entropy achieved by a split. Another indicator of impurity is the Gini index, which can be calculated using the equation :


(3) $$\begin{array}{l} Gini\left(S\right)=1-\overset{n}{\underset{i=1}{\sum }}p_{i}^{2} \end{array}$$

where *S* is the collection of examples and *p*_*i*_ is the percentage of examples that belong to class *i*. A few advantages of decision trees include their capacity to work with both category and numerical data, simplicity of interpretation, and capacity for handling missing values. However, some of the significant drawbacks of the decision tree are the propensity to overfit the training data and sensitivity to slight data changes.

**b) XGBoost model**

XGBoost (eXtreme Gradient Boosting) is a popular and efficient implementation of the gradient boosting algorithm for machine learning (Jiang et al., 2021). It is employed for supervised learning problems, i.e., classification and regression. In XGBoost, a decision tree is used as the weak learner, which is combined with other decision trees to create an ensemble model. The objective of XGBoost is to minimize the loss function, which can be expressed mathematically as:


(4) $$\begin{array}{l} Loss=\underset{\theta }{\min}\overset{n}{\underset{i=1}{\sum }}L\left(y_{i},{ŷ}_{i}\right)+\overset{T}{\underset{j=1}{\sum }}\Omega \left(f_{j}\right) \end{array}$$

where *i* and *j* are indexing variables used to sum overall training examples and decision trees in the ensemble respectively, θ represents the model parameters that we want to find in order to minimize the objective function and *n* is the number of training examples in our dataset. The difference between *y*_*i*_ (true label) and ŷ_*i*_ (predicted label) for each training example *i* is represented by *L*(*y*_*i*_, ŷ_*i*_). Accordingly, $\overset{n}{\underset{i=1}{\sum }}L\left(y_{i},{ŷ}_{i}\right)$ measures the sum of the loss function for overall training examples.

In addition, a regularization term Ω(*f*_*j*_) for the *j*-th decision tree is also used in the objective function. This helps to prevent overfitting which can occur when the model is too complex and fits the training data too closely. Given that *T* is the number of decision trees in the ensemble and *f*_*j*_ is the *j*-th decision tree in the ensemble, $\overset{T}{\underset{j=1}{\sum }}\Omega \left(f_{j}\right)$, i.e., the sum of the regularization term for overall decision trees in the ensemble measures the overall complexity of the model.

As shown in Equation (4), the objective function computes the overall loss function by combining both the loss term and regularization term. XGBoost utilizes gradient boosting to enhance the model during the training process by minimizing this objective function. It repeatedly applies a new weak learner to the residual errors of the previous iteration, adjusting the learner's parameters to minimize the objective function, until the function reaches a minimum or the maximum number of iterations is reached.

XGBoost has several features, such as parallel processing, optimized memory usage, and handling of missing values and sparse data, that make it an effective tool for large-scale machine-learning problems.

**c) Artificial Neural Network**

Artificial Neural Network (ANN) is a machine learning model based on the structure and function of the human brain (Zappone et al., 2019). It is composed of interconnected nodes or neurons that are connected to multiple other neurons through pathways or synapses. Each artificial neuron receives inputs from other neurons and performs a simple mathematical operation, known as *activation function*, on those inputs to produce an output signal. Activation functions are used in artificial neural networks to introduce non-linearity in the model and enable the network to learn complex relationships between input and output variables. This is important for achieving high accuracy in prediction tasks and for avoiding the problem of the vanishing gradient in deep neural networks. The activation function can be a threshold function, such as the step function, or a non-linear function, such as the sigmoid, the rectified linear unit (ReLU) function, Hyperbolic Tangent (Tanh) function, and Softmax (Karlik and Olgac, 2011).

The output signal from one neuron is then passed as input to other neurons in the next layer. The weights on the connections between neurons are adjusted during the training process using algorithms such as backpropagation. The training process involves presenting the ANN with a large number of input-output pairs, also known as training examples, and adjusting the weights so as to minimize the error between the model's predictions and the actual output. The error is calculated using a loss function. ANN layers can be mathematically represented as follows. Suppose *x* = [*x*_1_, *x*_2_, *x*_3_, …, *x*_*n*_] is the input vector and *n* is the number of inputs, the hidden layer output *h* can be defined as:


(5) $$\begin{array}{l} h=f\left(W\cdot x+b\right) \end{array}$$

where *h* is the hidden layer output, *f* is the activation function, *W* is the weight matrix, and *b* is the bias. After the weighted sum, an activation function is applied to introduce non-linearity into the model.


(6) $$\begin{array}{l} ŷ=g\left(h\right) \end{array}$$

where ŷ is the output after applying the activation function. This process continues throughout the layers in the neural network, and at the last layer, i.e., output layer, the discrepancy between the predicted and actual values is quantified via a loss function. Common loss functions include Mean Squared Error (MSE), Cross-Entropy, and Binary Cross-Entropy. Given *y* and ŷ are the target output and the predicted output, respectively, the loss function can be written as:


(7) $$\begin{array}{l} E=\frac{1}{2}\sum {\left(y-ŷ\right)}^{2} \end{array}$$

In the first phase of the forward pass, inputs are fed through the network, and the output of the network is computed using the current values of the weights and biases. Then, loss computation is computed as shown in Equation (8). Once the loss error is computed, backpropagation is carried out and the weights and bias are updated based on an optimization algorithm such as stochastic gradient descent algorithm, as shown in the following equations


(8) $$\begin{array}{l} \Delta W=-\alpha \cdot \frac{\partial E}{\partial W} \end{array}$$

(9) $$\begin{array}{l} \Delta b=-\alpha \cdot \frac{\partial E}{\partial b} \end{array}$$

where Δ*W* and Δ*b* are the changes in weight and bias, α is the learning rate, and $\frac{\partial E}{\partial W}$ and $\frac{\partial E}{\partial b}$ are the partial derivatives of the error with respect to weight and bias. Further, the forward pass, loss computation, and back propagation steps are repeated for a fixed number of iterations or until the loss function converges to a minimum.

### 3.3 Model-agnostic explainable AI methods

#### 3.3.1 SHAP

SHAP (SHapley Additive exPlanations) is a popular model-agnostic technique for explaining the output of any machine learning model (Mangalathu et al., 2020). It uses Shapley values from cooperative game theory that quantify the contribution of each player to a coalition. Specifically, it attributes the importance of each feature to the final prediction by calculating the contribution of each feature to the difference between the predicted value and a baseline reference value, assigning credit or blame to each feature based on how much it shifts the prediction away from the baseline.

In the context of feature attribution in machine learning, the Shapley value can be used to allocate the contribution of each input feature to the prediction of the model. In other words, the SHAP value of a feature represents the contribution of that feature to the difference between the actual output and the expected output of the model. Formally, the SHAP value of a feature for a specific instance *x* can be defined as shown in Equation (10).


(10) $$\phi_{i}(x)=\frac{1}{K}\sum_{S\subseteq N\{i\}}\frac{|S|!(K-|S|-1)!}{K!}(f(x_{S}\cup \{i\})-f(x_{S}))$$

where, ϕ_*i*_(*x*) represents the SHAP value of feature *i* for instance *x*. Note that *K* and *N* are the total number of input features and the set of all input features, respectively. *S* corresponds to the subset of *N* that does not contain feature *i*. The model prediction function is termed as *f*. Further, *x*_*S*_ is the instance with the features in *S* set to their expected values and *x*_*S*_∪{*i*} is the instance with feature *i* set to its actual value.

The SHAP values help to assign the contribution of each feature toward the model prediction with the help of summary plots, wherein the absolute SHAP scores rank the features by their importance. In addition to the global prediction, SHAP values also provide a local explanation for a given instance. It shows the influence of features contributes to the prediction and can be used to explain why a particular prediction was made. The SHAP-based explanations can help in diagnosing issues with the model, assessing the fairness of the model and comparing the feature importance of different models.

#### 3.3.2 LIME

LIME (Local Interpretable Model-Agnostic Explanations) is yet another post-hoc explanation technique for explaining ML models (Mishra et al., 2017). LIME justifications can increase user confidence in an AI system. The goal of LIME is to provide explanations that are both locally faithful to the model and interpretable to humans.

LIME generates a simpler, interpretable model called the “local surrogate model” around the prediction that it wants to explain. This local surrogate model is trained on a set of perturbed instances around *x* and is used to generate explanations by examining the feature importance values of the simpler model. In other words, LIME approximates the model locally using an interpretable model such as linear regression or decision tree and generates explanations by perturbing the input instance and observing the effect on the output of the model. The mathematical formulation of local surrogate models with interpretability constraint is expressed as in Equation (11).


(11) $$\begin{array}{l} \text{explanation}\left(x\right)=\arg\underset{g\in G}{\min}L\left(f,g,\pi_{x}\right)+\Omega \left(g\right) \end{array}$$

To explain a model's prediction for a particular instance *x*, LIME generates an explanation model represented by *g*, that minimizes a loss function *L*. This loss function evaluates how accurately the explanation model *g* approximates the prediction of the original model *f*. Note that *G* refers to the family of possible explanations (e.g., all possible linear regression models.) and proximity measure π_*x*_ corresponds to the vastness of the neighborhood around instance *x* that is considered for explanation. The regularization term Ω(*g*) corresponding to the model complexity is kept low to prefer fewer features.

LIME can be used to visualize the feature importance values in various ways, e.g., bar chart, to help users understand how different features contribute to the prediction. The LIME-based explanation can help users understand the reasoning behind the model's predictions and can be useful for debugging and improving the model.

### 3.4 Implementation details

The training and test data are divided in a 70–30% ratio, using the sci-kit library^6^. Further, the grid-search technique (Syarif et al., 2016) is used to find the optimal hyperparameters of the ML model which results in the most accurate predictions. The training data set is applied to the XGboost classification machine learning model, Decision tree classification model, and Neural Network Classifier models. XGboost employs binary cross-entropy optimization by default for binary classification and has a verbosity of 1.

Regarding the Artificial Neural Network classifier, the hidden layer sizes were set to 5 and the activation function was set to “Logistic.” Out of two different optimization functions SGD (Breuel, 2015) and Adam (Salem et al., 2022), the Neural Network classifier outperformed using Adam, hence it was set as the default model optimizer. To improve accuracy, more filtering of the basic data was also done and a few more features were eliminated. Eventually, there were 3,00,000 rows of “0”s and 75,000 rows of “1”s in the dataset. The binary class values in the dataset were made nearly equal by using the ADASYN oversampling technique (Rupapara et al., 2022).

### 3.5 Evaluation protocols

The model's performance is assessed using Accuracy, Precision, Recall, F1-score, and AUC-score metrics, obtained during training and testing. The aforementioned evaluation metrics are explained in detail below:

- **Accuracy**: Accuracy assesses how well a model performs in categorizing or predicting outcomes. It is defined as the proportion of the model's right predictions to all of its other forecasts. Formally,

(12) $$\begin{array}{l} Accuracy=\frac{\left(TP+TN\right)}{\left(TP+FP+TN+FN\right)} \end{array}$$

where *TP* is the number of “true positives” which are the instances where the model predicts the COVID-19 cases as severe when the true labels are actually severe; *TN* is the number of “true negatives” which are the instances where the model predicts the COVID-19 cases as non-severe when the true label is actually non-severe; *FP* is the number of “false positives” which are the instances where the model predicts the COVID-19 cases severe when the true labels are actually non-severe, and *FN* is the number of “false negatives,” which are instances where the model predicts the COVID-19 cases as non-severe when the true labels are actually severe.
In addition to the accuracy metric, we also evaluate “accuracy after resampling” in our study. This helps to evaluate the performance of a machine learning model on a dataset that has been altered by methods like oversampling or undersampling to resolve unbalanced class distributions. It gauges the percentage of cases in the resampled dataset that are correctly categorized.

- **Precision**: Precision calculates what percentage of the model's positive predictions are accurate, i.e., what proportion of all positive detections are severe cases. Mathematically, it is represented as:

(13) $$\begin{array}{l} Precision=\frac{TP}{\left(TP+FP\right)} \end{array}$$

- **Recall**: Recall measures the proportion of COVID-19 cases that are predicted to be positive among all instances that are actually positive. The recall is defined as:

(14) $$\begin{array}{l} Recall=\frac{TP}{\left(TP+FN\right)} \end{array}$$

This metric is referred to by other names such as True Positive Rate (TPR), Sensitivity, or Hit Rate.

- **F1-score**: It is a weighted average of the model's precision and recall, and it provides a single score that summarizes both of these metrics. The *F1* score can be calculated as follows:

(15) $$\begin{array}{l} F1=2*\frac{\left(Precision*Recall\right)}{\left(Precision+Recall\right)} \end{array}$$

- **AUC-score**: *AUC* (Area Under the Curve) is a commonly used metric to evaluate the performance of binary classification models. The *AUC* score represents the area under the Receiver Operating Characteristic (ROC) curve, which plots the True Positive Rate (*TPR*) against the False Positive Rate (*FPR*) at different classification thresholds. A higher *AUC* score indicates better performance of the classification model.

## 4 Experiments

We conduct two sets of experiments leveraging different AI models and XAI tools as mentioned in Section 3. First, the performance of AI models and their interpretations via the SHAP algorithm are instigated in ***Case study #1: SHAP***. Second, the same AI model's interpretation is analyzed using the LIME XAI interpretation model in ***Case study #2: LIME***. Both of the case studies are detailed in Sections 4.1 and 4.2, respectively.

### 4.1 **Case study #1: SHAP**

In this experimental analysis, three different ML models XGBoost (XGBoost), Decision tree (DT), and Artificial Neural network (ANN) are employed. After training the model, the model results along with the dataset are fed to the explainable SHAP function. The goal of SHAP is to explain the machine learning model's prediction by calculating the contribution of each feature to the prediction (Białek et al., 2022). Shapley values consider all possible predictions for an instance using all possible combinations of inputs. Because of this exhaustive approach, SHAP can guarantee properties like consistency and local accuracy. Due to the high time and resources usually taken for this model-agnostic method, SHAP values are often computed on a small subset of the data.

Based on the ML models, the method to compute SHAP values varies. For instance, exact SHAP values can be computed for tree models whereas only approximations are possible in other ML models using a linear regression mechanism. The SHAP function yields Shapley values which can be further used to generate various SHAP-based explainable plots. Some of the popular explainability plots are the SHAP summary plot, global bar plot, local bar plot, SHAP force plot, and waterfall plot. Direct comparison of SHAP values between models is not feasible due to scaling variations. However, it is possible to compare how various models weigh input features, by examining the shapes of different plots. The experiment results and explanatory analysis will be discussed in the Section 5.1.

### 4.2 **Case study #2: LIME**

Analogous to the study proposed using the SHAP algorithm in Case study #1, the results of ML models are analyzed and interpreted via another XAI tool known as Local Interpretable Model-agnostic Explanations (LIME) here. The same three ML models (XGboost, DT, ANN) are leveraged to evaluate the model performance. After training the model, the model results along with the dataset are fed to the LIME function. LIME builds sparse linear models around each prediction to explain how the black box model works in that local vicinity (Lundberg and Lee, 2017). LIME is actually a subset of SHAP but lacks the properties of consistency and accuracy. The LIME function yields an object which contains the values as in SHAP. LIME considers only local data for the graph yield. LIME is considerably faster compared to SHAP since it uses a simpler approach that generates local explanations by fitting an interpretable model to the data points in the vicinity of the input being explained. On the contrary, SHAP values involve computing a weighted average of feature attributions across all possible combinations of input features, which can be computationally expensive and time-consuming.

## 5 Experimental results

### 5.1 Analysis of ML models

The reliability of an interpretable AI result depends not only on the XAI tool but also on the right usage of the ML model used. To this end, we construct three different ML models as mentioned in Section 4, i.e., XGBoost (XGBoost), Decision tree (DT), and Artificial Neural network (ANN). We evaluated the model performances for the XGboost, Decision tree, and Neural Network models. The overall analysis of various ML model performances is shown in Table 2. Specifically, the performance of the three ML models is evaluated using accuracy, *F1*-score, precision, recall, and *AUC* metrics (refer Section 3.5).

**Table 2:** Performance analysis of AI models for COVID-19 severity prediction.

**S.No**.	**ML model**	**Accuracy**	**Accuracy after resampling**	**F1-score**	**Precision**	**Recall**	**AUC score**
1.	XGBoost classifier	83.583	86.895	0.859	0.877	0.842	0.845
2.	Decision tree (DT) classifier	81.817	83.234	0.8360	0.839	0.827	0.829
3.	Artificial neural network (ANN) classifier	79.396	81.159	0.7918	0.8015	0.7823	0.805

From Table 2, it can be observed that XGBoost outperforms the other models in terms of all evaluation protocols because of its ability to handle missing values, built-in regularization, parallel processing, ensemble learning, and gradient boosting. It surpasses other models with accuracy, and accuracy after resampling, *F1* score, precision, recall, *AUC* score values of 83.583, 86.895, 0.859, 0.877, 0.842, and 0.84, respectively. The performance of the decision tree model is found to be less than the XGBoost model because it does not employ an ensemble of decision trees, gradient boosting, and regularization. The performance of the Neural Network (ANN) model was also found to fall behind the other two models which may be ascribable to the lack of large data to achieve high accuracy, which would make it more difficult to train and optimize. As mentioned in Section 3.4, we tried with various hyperparameters such as different optimizers (SDG and Adam), changes in learning rates, and different numbers of epochs for ANN. Refer to Table 3 for the ANN result analysis for varying hyperparameters. The best model among the trials, i.e., a five-layered fully connected neural network with Logistic activation function and Adam optimizer with 200 epochs is chosen for our XAI analysis.

**Table 3:** Performance analysis of ANN models for different hyperparameters.

**S.No**.	**No. of layers**	**Optimizer**	**epoch**	**Accuracy (%)**
1.	3	SDG	50	76.05
2.	4	SDG	50	76.2
3.	4	Adam	200	77.34
4.	5	SDG	50	77.9
5.	5	SDG	200	78.2
5.	5	Adam	50	79.18
6.	**5**	**Adam**	**200**	**79.396**
7.	6	SDG	50	78.89
8.	6	Adam	200	79.34
9.	10	86.895	200	78.9

The best model is shown in bold letters.

### 5.2 Case study result#1: SHAP

As discussed in Section 4.1, SHAP-based result analysis is carried out in this section. SHAP is a model-agnostic technique that provides individual feature importance. It ensures consistency and fairness in the attributions and can provide both global and local explanations of the model's behavior on the severity-prediction task. In particular, the interpretations are drawn out using various explainability plots such as SHAP global bar plot, local bar plot, beeswarm plot, force plot, and waterfall plot. We analyse and compare the results of the SHAP algorithm for three different models, i.e., XGBoost, Decision tree, and Neural network models, and discuss its interpretations.

#### 5.2.1 Global bar plot

A SHAP-based global bar plot is a visualization technique that shows the impact of each feature on a model's output using SHAP (SHapley Additive exPlanations) values. The bar plot displays the average absolute SHAP value for each feature, indicating its relative importance in the model. This plot explains the contributions of each feature with respect to the whole data therefore this plot is called the SHAP Global bar plot. The bar length implies the average impact of the individual features on the models' output. The features are listed top-down with their decreasing importance.

**Figure 2:**
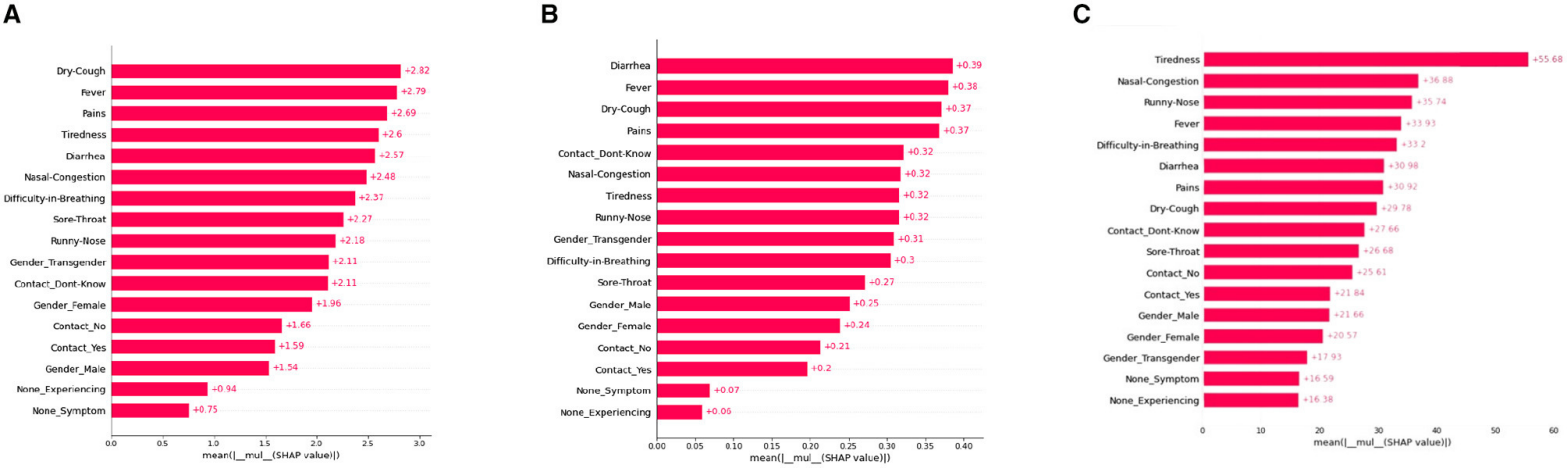
SHAP global bar plots for various ML models. **(A)** XGboost, **(B)** decision tree, **(C)** artificial neural network.

According to the interpretations depicted in Figure 2, some features such as “Dry-cough,” “Tiredness,” “Fever,” “Nasal-congestion,” and “Diarrhea,” are found to be the common important features (among the top five bar) as per XGBoost, Decision tree, and ANN models. On the other hand, the least mean SHAP value was found to be “None symptoms” [sic] for XGboost and “None Experiencing” for the decision tree and ANN models, respectively.

#### 5.2.2 Local bar plot

**Figure 3:**
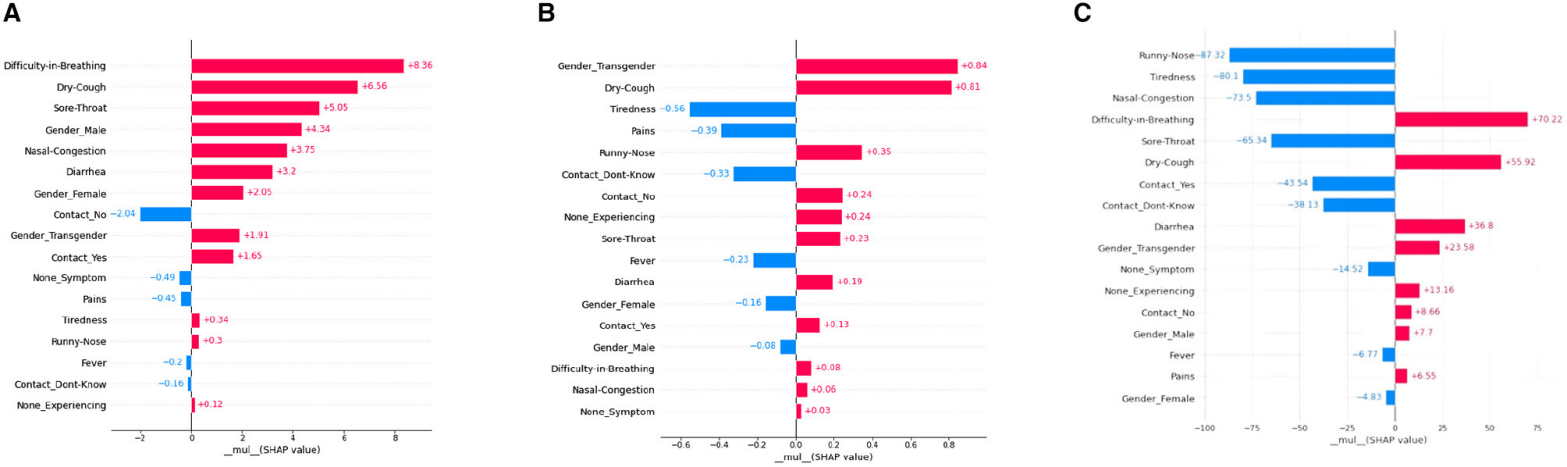
SHAP Local bar plots for various ML models. **(A)** XGboost, **(B)** decision tree, **(C)** artificial neural network.

A SHAP-based local bar plot is a technique for interpreting the feature importance of an individual prediction made by a machine learning model. It uses SHAP values to quantify the contribution of each feature to the prediction of a particular patient. See Figure 3 for the representation of our SHAP local plots. Such a plot is helpful in comprehending the contribution of each feature to the final prediction for a single instance or observation in the dataset. The local bar plot visualizes the SHAP values for each feature in a horizontal bar chart. The length of each bar indicates the magnitude and direction of the feature's impact on the prediction. Positive and negative values on the x-axis show whether the feature is contributing to a higher or lower prediction, respectively. The color of each bar represents the value of the feature for the instance being explained, where red indicates high values and blue indicates low values.

**Figure 4:**
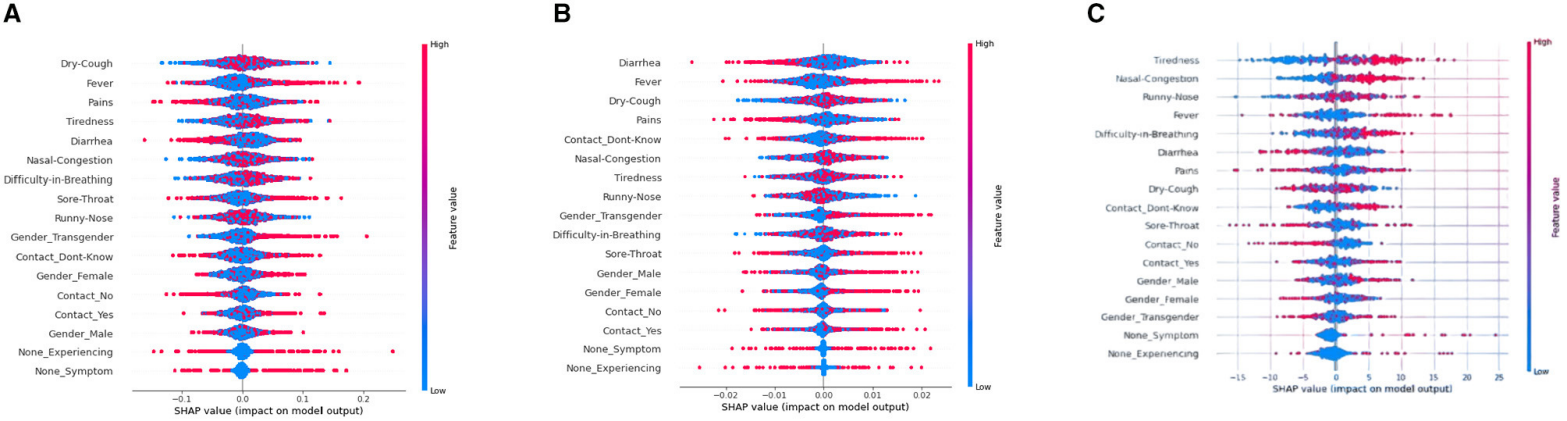
SHAP Beeswarm plots for various ML models. **(A)** XGboost, **(B)** decision tree, **(C)** artificial neural network.

According to interpretations from the Figure 4 for a particular patient, “Difficulty in Breathing” has the highest positive mean SHAP value, and “Contact-No” has the highest negative mean SHAP value for the model XGBoost. The decision tree model's local plot shows “Gender-Transgender” has the highest positive mean SHAP value for that particular patient and “Tiredness” has the highest negative SHAP value. For the ANN model, “Difficulty-in-Breathing” has the highest positive SHAP value, and “Runny-Nose” has the highest negative SHAP value. Additionally, the importance of “Dry-Cough” is found to be predominant in all three models (XGBoost, Decision Tree, ANN model), interpreting the significance of that feature in the considered patient.

#### 5.2.3 Beeswarm plot

Referring to Figure 4, SHAP Beeswarm plots are represented to identify patterns and relationships between the features and the model's predictions. A beeswarm plot is a type of scatter plot that is used to visualize the distribution of a single continuous variable or multiple continuous variables. The SHAP values of each feature for each instance in the dataset are represented as a vertical line or “bee.” Each dot or bee on the plot represents the SHAP value for a single row and a data feature. The plot is sorted by the feature's absolute SHAP value, with the most significant features at the top. The density of the bees or points in a particular region represents the concentration of data points in that region. Beeswarm plot helps to see the spread of SHAP values for each feature to understand how much variability there is in the feature's impact across the dataset, and whether there are any outliers or unusual cases.

The Figure 4 show that the points/bees for the top feature “Dry-cough,” “Runny-Nose,” “Tiredness,” “Nasal congestion,” and “Difficulty-in-Breathing” are more accumulated near the median line for XGBoost, Decision tree, and ANN model. On the contrary, the least accumulated points, i.e., the least denser feature are found to be “None-Symptom” and “None-Experiencing” for XGBoost, Decision tree, and ANN model.

#### 5.2.4 Force plot

**Figure 5:**
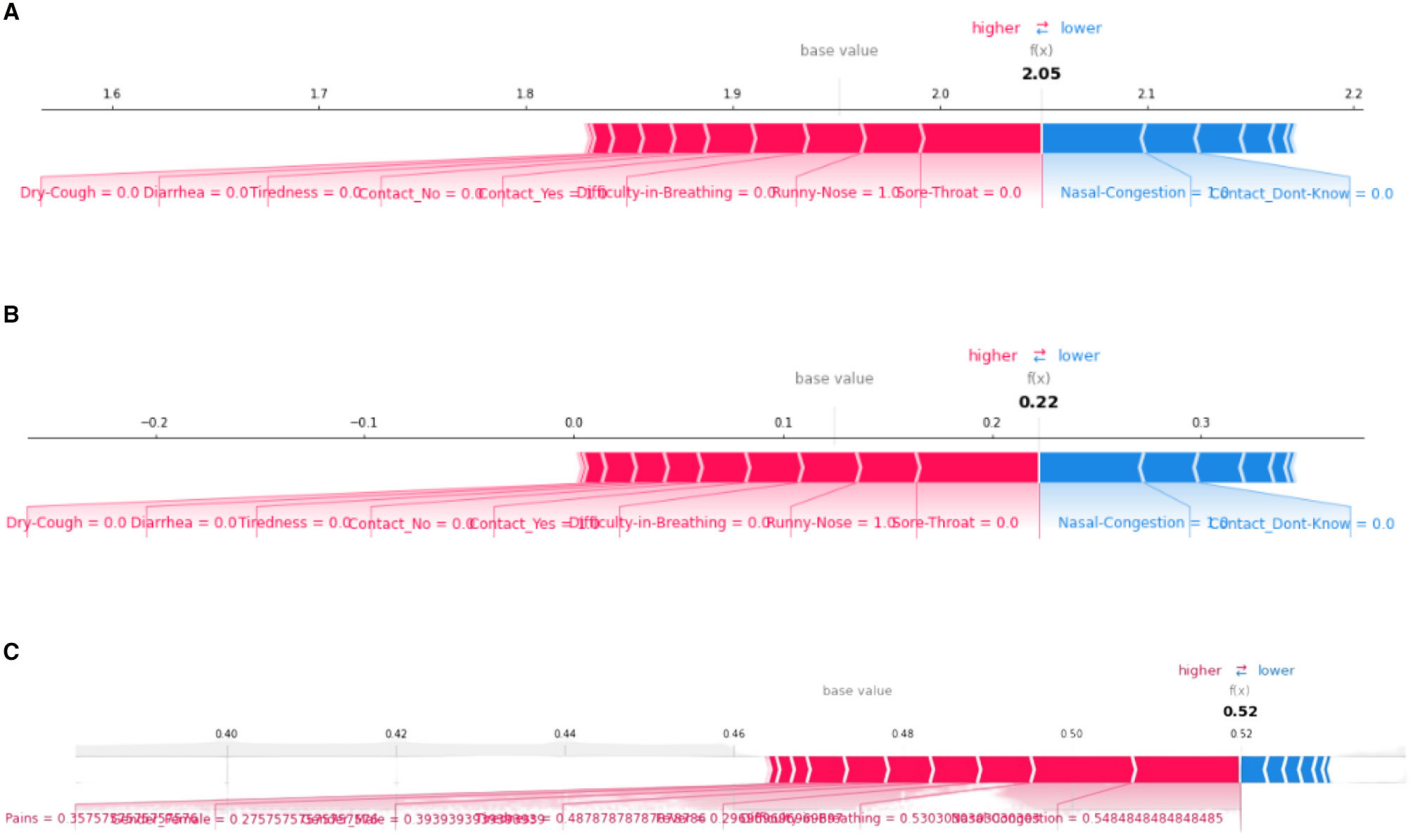
SHAP force plots for XGboost **(A)**, decision tree **(B)**, and artificial neural network **(C)** model, respectively.

Another very important SHAP-based XAI tool is the Force plot. It also shows how each attribute contributes to a machine-learning model's output for a selected patient. However, in this plot, each feature's significance is measured using SHAP values, along with the direction and size of its influence on the prediction. Refer to Figure 5 for our force plot results. From Figure 5, it is observed that “Difficulty-in-Breathing,” “Sore-Throat,” and “Runny-Nose” impart high positive contributions toward COVID severity, which is in alignment with the common clinical sense too. Similarly, for that particular patient, “Nasal-Congestion,” “Contact-No,” and “Contact-don't-Know” showed negative contribution.

Referring to Figure 5, the base value of f(x) represents the predicted outcome of the model when all the input variables are set to their reference or baseline values. It is represented by a vertical line at the center of the plot and is typically the mean or median value of the input variables from the training data. The base values of the plots are at 2.05, 0.22, and 0.52 for XGBoost, decision tree, and ANN model, respectively. In the decision tree and XGBoost models, the top positive contributing features are “Runny-Nose” and “Sore-Throat” whereas for the ANN model, the top positive contributing features are “Nasal-Congestion” and “Difficulty-in-Breathing.”

#### 5.2.5 Waterfall plot

**Figure 6:**
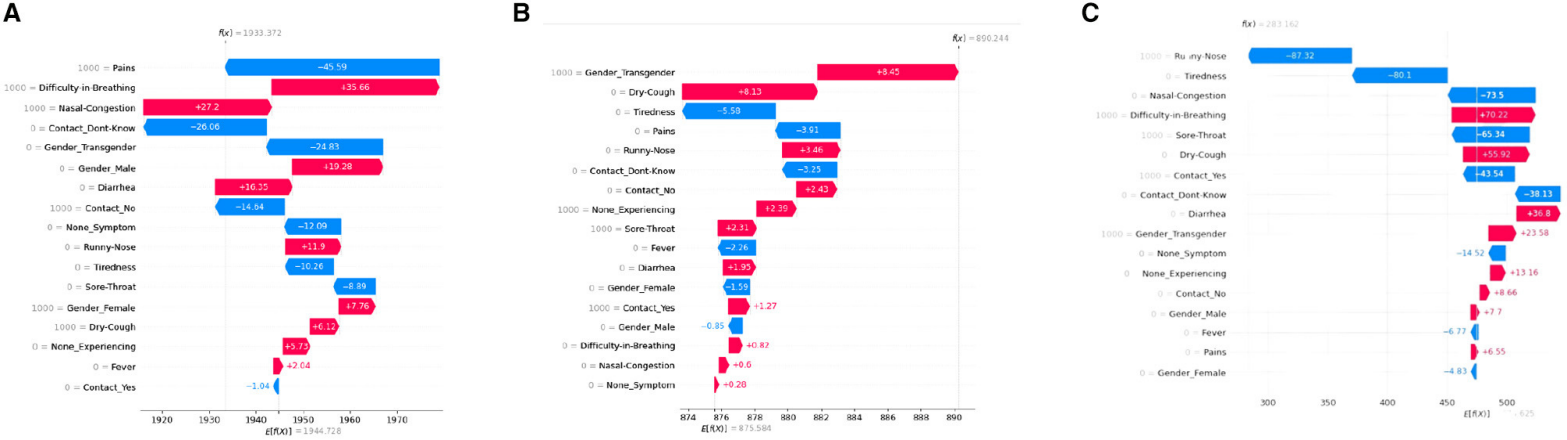
SHAP waterfall plots for various ML models. **(A)** XGboost, **(B)** decision tree, **(C)** artificial neural network.

A SHAP waterfall plot visualizes the individual and collective contributions of features to a model's prediction using Shapley values. Such a plot helps to understand how the predicted value for a particular instance deviates from the model's base value, and how each feature contributes to this deviation. See Figure 6 for our SHAP waterfall plot.

Waterfall charts are designed to present explanations for specific forecasts, hence they demand a single row of an explanation object as input. The bottom of a waterfall plot starts as the expected value of the model output, and then each row displays how the positive (red) or negative (blue) contribution of each feature shifts the value from the expected model output across the background dataset to the model output for this prediction. The units on the x-axis are log-odds units, so negative values imply probabilities of <0.5.

The baseline prediction is the initial predicted value before taking into account any of the feature contributions. It is represented by the central zero line on the x-axis. We could infer that “Difficulty-in-breathing,” “Gender-Transgender,” and “Difficulty-in-breathing” are the highest positively contributing features for the models XGBoost, decision tree, and Neural network respectively for the selected patient.

### 5.3 Case study result #2: LIME

In this section, the result analysis of the Local Interpretable Model-agnostic Explanation (LIME) XAI tool, as described in Section 4.2, is carried out. LIME is a local explanation technique, i.e., it focuses on explaining the prediction of a single instance at a time. Since LIME assumes that the model is locally linear, it generates a local model to approximate the behavior of the black box model around the instance of interest, and then uses the local model to generate explanations for that instance. Because of this, it is not possible to generate global plots as in SHAP, to provide a global understanding of the model's behavior across the dataset. Instead, LIME provides local explanations, giving some insight into how the model behaves around a specific instance. In particular, the results are interpreted via LIME explainability tools such as local bar plots and violin plots.

#### 5.3.1 Local bar plot

LIME is model-agnostic, making it flexible and powerful for local model interpretation, providing insights into how the model is behaving for a specific instance. LIME local bar plot proves to be more efficient than the SHAP algorithm when it comes to Local interpretations. A LIME-based local bar plot shows the contribution of each feature to the prediction for a specific instance or sample, with bars colored according to their sign. The most important features are shown at the top and the least important at the bottom.

**Figure 7:**
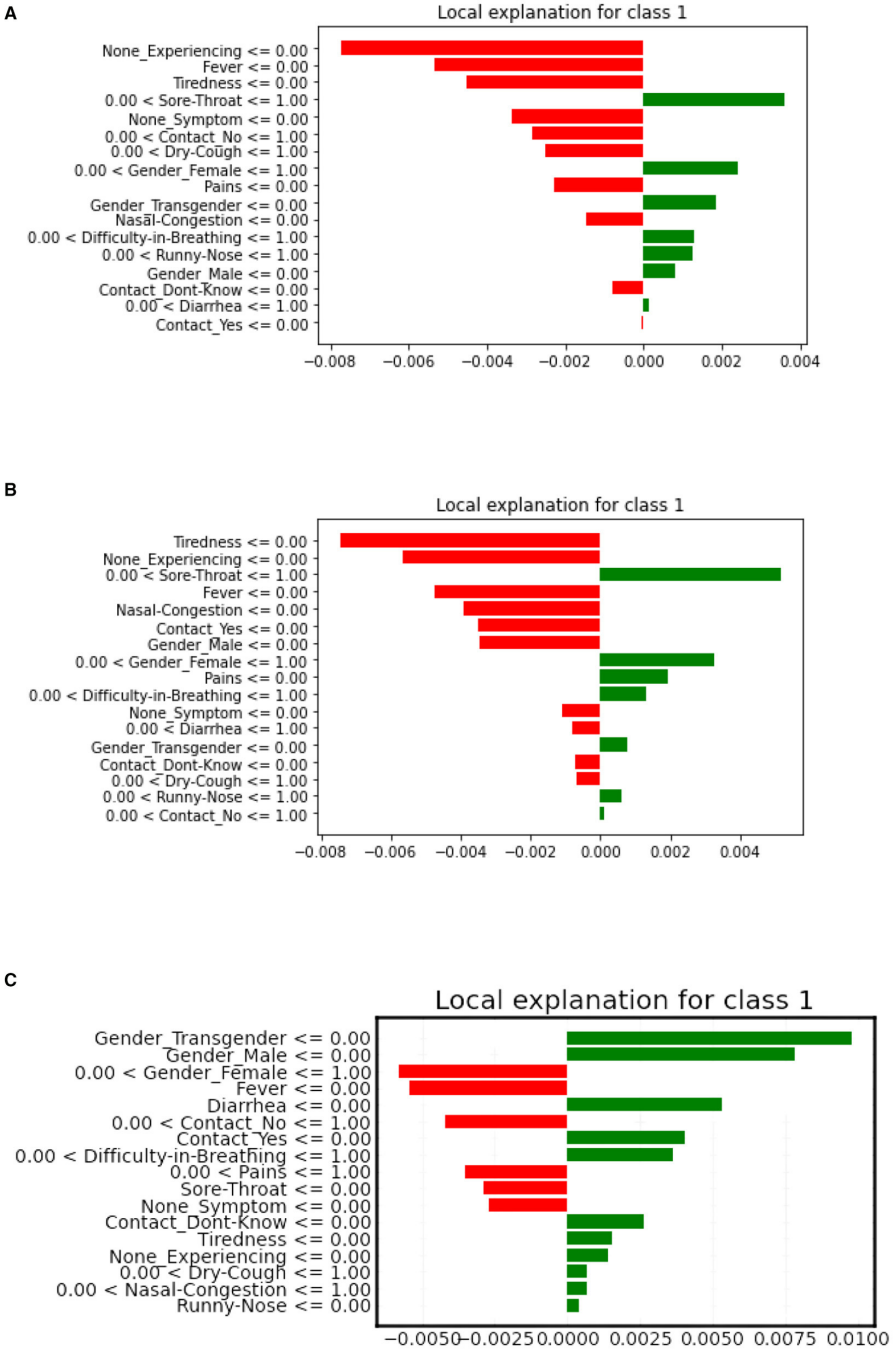
LIME local bar plots for XGboost **(A)**, decision tree **(B)**, artificial neural network **(C)** model, respectively.

Refer to Figure 7 for the LIME local plot visualization, for a specific patient observation in our COVID dataset. It is evident from the figure that the features “Sore-throat” and “Pains” have the highest positive contribution to the selected patient, according to the XGBoost and Decision tree plots. The ANN-based LIME plot deviates a bit in interpretation, highlighting “Gender-transgender” as the high-impact feature.

#### 5.3.2 Violin plot

Yet another LIME-based explainability plot is the violin plot. The violin plot represents the distribution of feature importance values generated by LIME for a given instance. Each feature is represented by a vertical line or “violin” that shows the distribution of its important values across multiple samples generated by LIME. The width of the violin indicates the density of the distribution, with wider parts indicating more frequent importance values.

**Figure 8:**
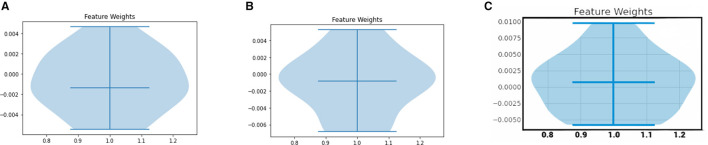
LIME Violin plots for various ML models. **(A)** XGboost, **(B)** decision tree, **(C)** artificial neural network.

The LIME Violin plots for the COVID Severity class for a sample instance in our COVID dataset are depicted in Figure 8. Note that it represents the cumulative LIME value analysis for all features, per person. The middle line represents the median, the top line represents the top range, and the bottom line represents the bottom range of the LIME values. In the XGBoost model, the spread of feature weights for the COVID severity class are high in the regions above below, and around the median line. In the decision tree model, the spread of feature weights is high in the median region. In the ANN model, the spread of feature weights are high in the below and median regions.

In order to summarize all the aforesaid result analysis based on SHAP and LIME, all the key findings from those XAI tools are consolidated as a table in Table 4.

**Table 4:** Summary chart showing the findings from the XAI tools.

**Visualization plot**	**XAI tool**	**Global/local**	**Result and key symptoms**	**Medical significance**
Global bar plot	SHAP	Global	“Dry-cough,” “Tiredness,” “Fever,” “Nasal-congestion,” and “Diarrhea”	Overall model behavior; Feature importance ranking;Magnitude of contribution;Verification of domain knowledge
Local bar plot	SHAP	Local	For a selected patient, “Difficulty in Breathing” is found	Explanation of individual predictions; Positive and negative contributions; Feature impact magnitude.
Beeswarm plot	SHAP	Global	“Dry-cough,” “Runny-Nose,” “Tiredness,” “Nasal congestion,” and “Difficulty-in-Breathing”	Interaction effects among features;Outlier detection;Distribution of contributions.
Force plot	SHAP	Local	Positively contributing features for a selected patient are “Runny-Nose” and “Sore Throat”	Granular explanation of an instance-specific breakdown of feature contributions; Net impact calculation indicating the total impact of all features on the prediction.
Waterfall plot	SHAP	Local	For the selected patient, “Difficulty in Breathing” is observed	Visualizing Cumulative Impact of how individual components contribute; Identifying Key Drivers; Forecasting and planning.
Local bar plot	LIME	Local	“Sore-throat,” “Difficulty-in-Breathing,” and “Pains” as the highest positive contribution to the selected patient	Explain individual predictions; Feature impact magnitude (both positive and negative); Model consistency; Verification of domain knowledge.
Violin plot	LIME	Local	Overall feature importance spread per patient	Depicting the distribution of feature importance values generated by LIME; density of the distribution indicates frequent importance values.

## 6 State-of-the-art comparison

In this section, a comparative analysis of our work against state-of-the-art works is carried out. The holistic summary report of the analysis is depicted in Table 5. From the chart, it can be observed that Barda et al. (2020) presented a SHAP-based mortality prediction model on Israel's COVID-19 patients. In that work, a decision-tree-based gradient boosting model was employed and SHAP scores were used to comprehend the contribution and effect of the selected features such as age, Chronic respiratory disease, hospitalization duration, Ambulance services count etc. Yet another similar work by Snider et al. (2021) explored the use of the XGBoost AI model and SHAP explainability tool to study COVID-19 instances of patient fatalities in Ontario.

**Table 5:** State-of-the-art comparison of XAI models on various COVID-19 datasets.

**S.No**.	**References**	**Data**	**Region**	**Model**	**Result**
1.	Barda et al. (2020)	Clalit Health Services' (CHS) COVID-19 patient cohort	Israel	COVID-19 mortality risk prediction model	AUROC 0.820; SHAP analysis shows the impact of age, hospital duration, and other attributes such as Chronic respiratory diseases, diabetes etc.
2.	Snider et al. (2021)	Ontario Health Data Platform	Ontario, Canada	mortality predictions	XGBoost AUC 0.956; SHAP-based analysis shows the highest importance of variables for mortality, i.e., age, date of test, sex, and presence/absence of chronic dementia.
3.	Ong et al. (2021)	X-Ray images with different conditions, taken from the COVIDx dataset	Multinational cohort	diagnosis of COVID-19 infection	SHAP & LIME on X-ray images indicating the significant lung region significant for COVID prediction
4.	Zou et al. (2022)	AI predictive model known as the Community-Acquired Pneumonia and COVID-19 AI Predictive Engine (CAPE)	Singapore	AI predictive model for Pneumonia and COVID-19	AUC of 0.803; Ensemble XAI, which is based on the SHAP and Grad-CAM++ methods, provides a visual explanation for a deep learning prognostic model that predicts the mortality risk.
5.	Kumar et al. (2022)	COVIDx dataset	Multinational	Convolutional neural network based COVID-19 prediction	Grad-CAM applied on top of SARS-Net CNN and GCN models for visual interpretations
6.	Rahimi et al. (2023)	Patients with positive polymerase chain reaction test for COVID-19	Quebec, Canada	Deep forest/XGBoost ML models for severity prediction	Explainable approaches such as LIME, SHAP, PIMP, and anchor; Correlation with diabetes and dementia is found out
7.	Gabbay et al. (2021)	Open dataset provided by the Mexican Federal Health Secretary through the General Director of Epidemiology	Mexico	MLP and RF decision trees for prediction	LIME-based explainable model; Individual-specific local explanations.
8.	Ours	Custom-made dataset collected from publicly available COVID-19 Indian datasets from various resources	India	COVID-19 Severity Prediction and Symptom analysis	AUC 0.869 for XGBoost; LIME, SHAP based extensive global and local analysis on Indian dataset).

Other than numerical data-based mortality prediction, image-based COVID-19 diagnosis was also addressed in some of the literature, such as in Ong et al. (2021), Kumar et al. (2022), and Zou et al. (2022), using chest X-ray images. Using SHAP, LIME, and GradCAM helps clinicians in the disposition and severity assessment of COVID/pneumonia cases visually by showing the area of interest, thereby increasing the transparency and the interpretability of the model. The key findings of those papers are also summarized in Table 5.

In contrast to the aforesaid works, i.e., mortality prediction/COVID diagnosis, we present COVID-19 severity Prediction and symptom analysis using XAI models leveraging numerical data, which is not much explored in the literature. One study (Rahimi et al., 2023) presented the use of explainable machine learning models to predict COVID-19 severity among older adults in the province of Quebec, Canada. In that work, the correlation between different variables such as diabetes and dementia, and the severity of COVID-19 in the older adult population was discovered. Another work from Gabbay et al. (2021), combined the machine learning models with a LIME-based XAI model to provide local explanations on patients' severity prediction, which used the dataset provided by the Mexican Federal Health Secretary. In contrast to the aforementioned works, our work not only presents a more extensive analysis of both global (SHAP-based analysis on a holistic dataset) and local models (individual result analysis) for severity prediction on the cross-section in the Indian population but also facilitates “symptom analysis.” To the best of our knowledge, “XAI-for-COVID symptom analysis” works are not encountered in the literature.

Furthermore, no similar works using XAI for COVID analysis are held in Indian datasets. The only available work using XAI in the Indian database is by Pandianchery et al. (2022). However, it addresses the task of predicting COVID-19 cases in different provinces of India, using a Recurrent Neural Network (RNN) based model. Hence, our work marks the first work leveraging XAI tools for COVID-19 data analysis for severity prediction and symptom analysis in the Indian patients dataset.

## 7 Discussion

### 7.1 Significance

AI-powered medical analysis falls in one of the ascendent fields of scientific research that requires immediate scientific attention. Explainable AI tools in healthcare are crucial for ensuring transparency, trustworthiness, and accountability in the decision-making process of AI systems, ultimately leading to better patient care.

Our work on using “Explainable Artificial Intelligence Tools for COVID-19 Severity Prediction and Symptom analysis” helps to understand the impact of attributes and symptoms that cause severe COVID-19 and provide clinicians with an intuitive understanding and interpretability of the impact of risk factors in the model. We hypothesize that our model can proactively analyse and predict future similar COVID-like scenarios in light of the key findings of this research work. Integrating such interpretable AI models with clinical observations, we contemplate that medical professionals can make timely decisions and also prevent/notify high-risk members based on the symptoms. Furthermore, the methodology and results obtained in our COVID-19 study may be extended to other medical conditions as well, e.g., other respiratory diseases, stroke, etc. Such an explainable computer-aided tool will be highly beneficial for medical practitioners in validating their decisions and expanding the knowledge base with the help of AI. Such an AI and human-in-the-loop model adaptively amalgamates human knowledge as well as AI tools thus bridging the existing semantic gap between man and machine and can instill new interests in the multi-disciplinary research community of AI and medicine (Bakken, 2023).

### 7.2 Limitations of the work

First, our study is based on the custom-made dataset collected from various available COVID-19 data repositories in India, as referred to in Section 2. However, it lacked external validation by an independent cohort, which could provide further evidence to confirm the superiority of the proposed prediction model. We believe that the current study could be further expanded by including related data from different regions and/or countries for external validation. Furthermore, more detailed research also by incorporating relevant clinical co-morbidity risk factors, environmental factors, lifestyles, and other factors would also help in improving future predictions and examining the impact of confounding factors.

The interpretation analysis of model-agnostic XAI tools SHAP and LIME clearly suggests that certain models can be good at one aspect and still may be suboptimal in others. It can be observed that a better model in terms of AUC may not imply the most accurate model in terms of medical theory and vice versa. For instance, although the ANN model has the worst AUC among the ML models (refer Table 3), it was found from the SHAP individual force plot that only the ANN model considered “Nasal Congestion” while making a prediction, even when the others were not. Similarly while analysing via SHAP local bar plot/waterfall plot, some attribute, i.e., “Gender_Transgender” was assigned the highest weightage by the Decision tree model, in contrast to the XGBoost and ANN models that assigned “Difficulty-in-breathing” as the major attribute. Nonetheless, from a medical perspective, the latter observation on breathing makes a more meaningful observation than the former gender cue, considering the patient's COVID severity condition. The reason behind such deviations may be ascribable to the combination of the number of data samples involved in the study, selection of feature values, preprocessing techniques, and hyperparameter tuning. Although examining these aspects can yield valuable insights, it falls beyond the scope of this project.

Similarly, although SHAP and LIME facilitate model interpretation, the choice between them depends on the specific use case, the nature of the model, and the desired level of explanation (SHAP provides both global and local explanations whereas, LIME focuses on local explanations.). SHAP leverages Shapley values to find the contribution for each feature across different predictions, whereas LIME leverages a surrogate model (e.g., a linear model) to approximate the model's behavior locally. Further, SHAP is more stable and consistent and can be computationally intensive, whereas LIME is more sensitive to the choice of perturbations AND computationally lighter. Due to these differences, we noticed that different XAI models could provide different interpretations as well. In this regard, we believe that some ensemble/hybrid models or using multiple techniques in tandem also could be explored for a more comprehensive understanding of a machine learning model's behavior.

### 7.3 Future directions

Referring to future works, we believe that there is a lot of room to be explored within the XAI paradigm. In this work, we utilized only the numerical database for COVID symptom analysis and severity prediction. However, we contemplate that utilizing other modalities could augment the overall performance of the proposed model. For instance, additional electronic health records and medical imaging data (chest imaging X-ray/CT scan, etc.) can provide a comprehensive picture of the patient's health status as well as integrate with clinical decision support systems (CDSS). We foresee that such additional data sources and active learning by collaborating with healthcare professionals can aid significantly in improving the accuracy and interoperability of the models. Further studies are needed to incorporate these models into a decision support system (e.g., web/mobile application) that aids in the handling of diseases like COVID-19 for primary healthcare providers and medical staff worldwide. This research will also assess their practicality and effectiveness within this context.

As pointed out in the previous section, it is found that there exists a semantic gap between the practical use of AI in medicine and clinical decisions. We foresee that incorporating explainable computer-aided tools in medicine can complement the medical practitioners in validating their decisions in a better interpretable way by facilitating “AI-&-human-in-the-loop” thus fusing the best of both worlds (Bakken, 2023). More research in this direction is expected in the future.

For the performance analysis, only the performance metrics and visualizations deemed most appropriate are found to be utilized. In addition, the comparisons between different XAI models have also not been much explored. To this end, the reliability of the XAI scores can be investigated at subject level by assessing the intra-consistency of the XAI scores and across subjects by analyzing the inter-similarity of the scores as done in Lombardi et al. (2021). This could be a relevant area for further research analysis and future works. In addition, we contemplate incorporating more diverse and representative datasets to improve accuracy and generalizability, developing sophisticated feature selection techniques to improve interpretability, and integrating multiple machine learning models to enhance the system's overall accuracy and robustness.

## 8 Conclusions

In this work, we proposed the application of a model-agnostic explainable artificial intelligence (XAI) framework to provide accurate explanations of machine learning algorithms and feature importance for medical output predictions. We adopted a cohort of COVID-19-affected patients within India as our dataset for the severity prediction and symptom analysis task. Three different machine learning models, i.e., XGBoost, decision tree, and Artificial Neural network models were applied to the data and the performances were analyzed after pre-processing. The XGBoost model is found to be performing the best because of its ability to handle missing values, built-in regularization, parallel processing, ensemble learning, and gradient boosting.

Further, to explore the feature's importance and its contribution to the predicted output, we leveraged two major XAI tools in this work. In particular, SHapley Additive exPlanations values (SHAP) and Local Interpretable Model-Agnostic Explanations (LIME) are utilized extensively to comprehend the importance of features and to provide better insights into model decision-making. The SHAP and LIME values were calculated for each of the models to interpret the model's outcomes. Extensive analysis in terms of visual representations, i.e., global bar plot, local bar plots, beeswarm plot, force plot, waterfall plot, and violin plot are conducted. The SHAP model gives local as well as global level plots for interpretations whereas LIME, as the name suggests, provides explanation at the local level only.

According to the SHAP values, the features “Dry-Cough,” “Tiredness,” “Fever,” “Nasal Congestion,” “Diarrhea,” and “Difficulty in Breathing” were found to be the most important symptoms in COVID-19 Indian patients, by all three models. This is found to be in consensus with the medical reports in India. Further, for different patient instances, local interpretations were also drawn out using local analysis tools of SHAP and LIME. Despite LIME's superior local explanation, SHAP interpretation is preferred due to its more solid theoretical foundation, and capacity for both global and local interpretability, consistency, and robustness. In the future, we envisage comparing the reliability of the XAI scores also at a subject level by assessing the intra-consistency of the XAI scores and across subjects by analyzing the inter-similarity of the scores.

## Data availability statement

The original contributions presented in the study are included in the article/Supplementary material, further inquiries can be directed to the corresponding author.

## Ethics statement

Written informed consent was not obtained from the individual(s) for the publication of any potentially identifiable images or data included in this article because Our case study does not involve any identifiable data. All the data we used are anonymized and contain binary features and targets showing the attributes and the corresponding presence of COVID. No individual data/identifiable information is involved in this work, hence written informed consent is not obtained.

## Author contributions

AN: Conceptualization, Formal analysis, Investigation, Methodology, Project administration, Supervision, Validation, Visualization, Writing—original draft, Writing—review & editing. HS: Data curation, Investigation, Software, Writing—original draft, Formal analysis. SS: Data curation, Investigation, Software, Writing—original draft, Formal analysis.

## Footnotes

^1^ https://www.who.int/publications/m/item/weekly-epidemiological-update-on-covid-19—22-march-2022

^2^Sample COVID-19 Indian dataset used in our study is available in https://github.com/NambiarAthira/COVID-XA.

^3^ https://github.com/IBM/covid19-india-data

^4^ https://www.kaggle.com/datasets/hemanthhari/symptoms-and-covid-presence

^5^We have consolidated our study data from multiple data repositories, including government resources, as mentioned in Section 3.1.1. All the data resources have sorted gender into either “Male,” “Female,” or “Transgender.” Hence, following the standard practice of representation of genders, we also used the same categories in this study.

^6^ https://scikit-learn.org/
