# Supplementary figures and images for: Model-agnostic explainable artificial intelligence tools for severity prediction and symptom analysis on Indian COVID-19 data
Source: Front Artif Intell. 2023 Dec 4;6:1272506. doi: 10.3389/frai.2023.1272506 (PMC10726049; doi:10.3389/frai.2023.1272506)

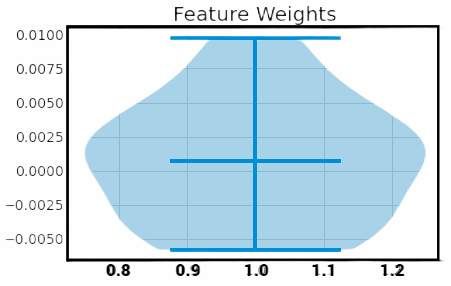

Supplement: Supplementary file 1 [file Data_Sheet_1.ZIP › Fig9c)lime_ANviolin.jpg]

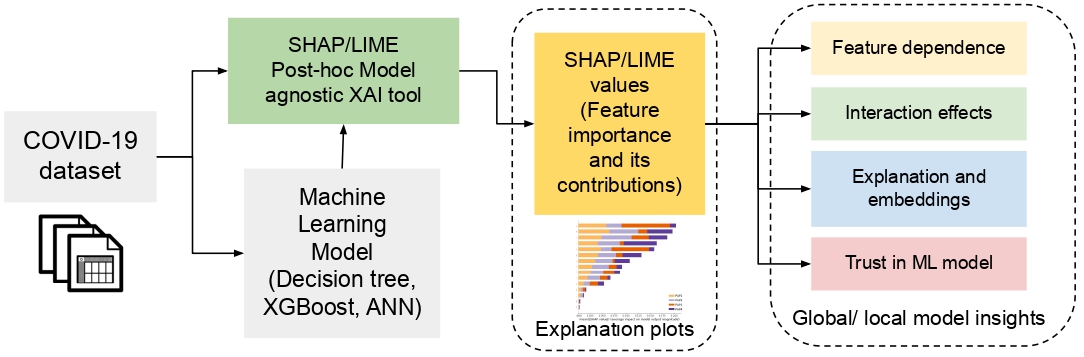

Supplement: Supplementary file 1 [file Data_Sheet_1.ZIP › Fig1)XAI-3.jpg]

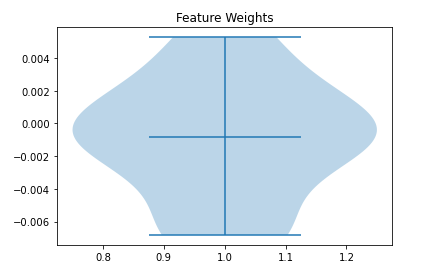

Supplement: Supplementary file 1 [file Data_Sheet_1.ZIP › Fig9b)lime_DTviolin.jpg]

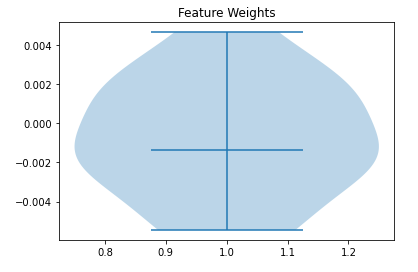

Supplement: Supplementary file 1 [file Data_Sheet_1.ZIP › Fig9a)lime_XGviolin.jpg]

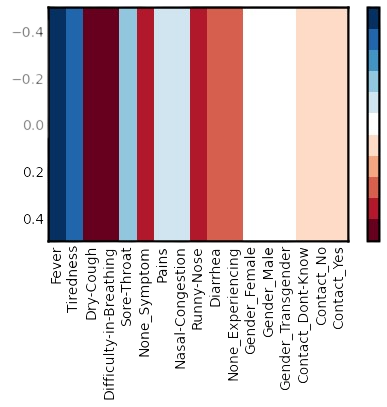

Supplement: Supplementary file 1 [file Data_Sheet_1.ZIP › Fig8c)lime_ANheatmap.jpg]

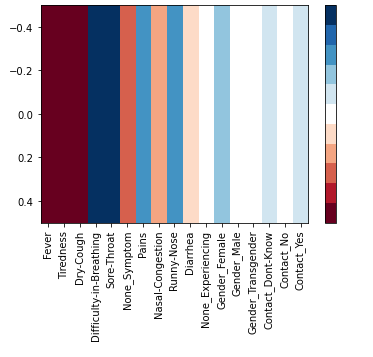

Supplement: Supplementary file 1 [file Data_Sheet_1.ZIP › Fig8b)lime_DTheatmap.jpg]

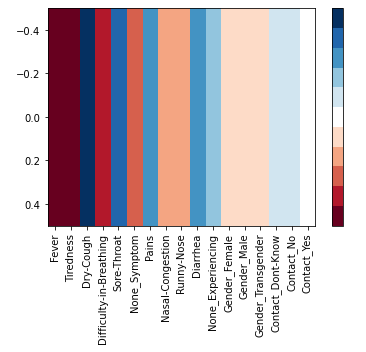

Supplement: Supplementary file 1 [file Data_Sheet_1.ZIP › Fig8a)lime_XGheatmap.jpg]

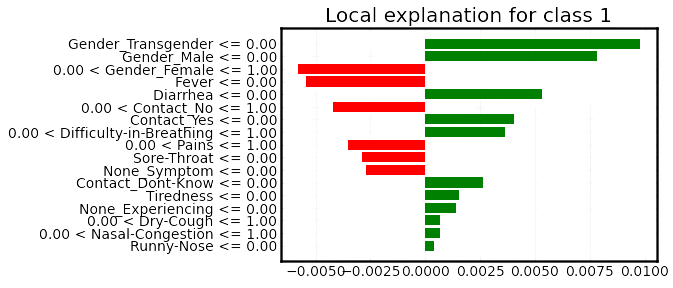

Supplement: Supplementary file 1 [file Data_Sheet_1.ZIP › Fig7c)lime_ANlocal.jpg]

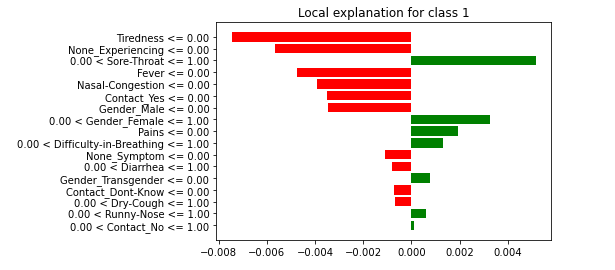

Supplement: Supplementary file 1 [file Data_Sheet_1.ZIP › Fig7b)lime_DTlocal.jpg]

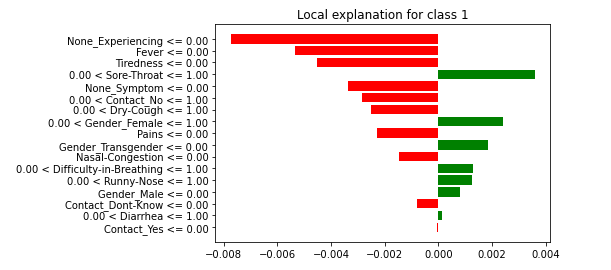

Supplement: Supplementary file 1 [file Data_Sheet_1.ZIP › Fig7a)lime_XGlocal.jpg]

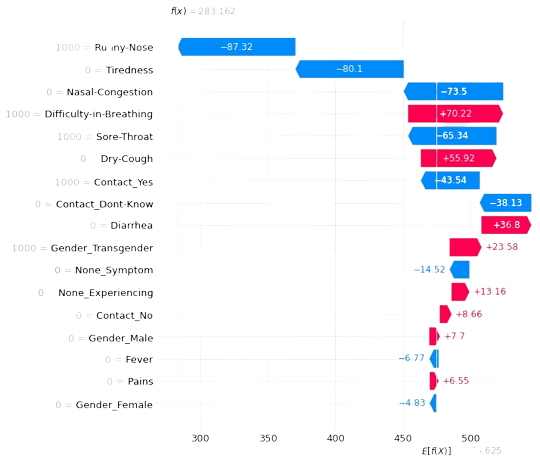

Supplement: Supplementary file 1 [file Data_Sheet_1.ZIP › Fig6c)ANN_waterfall.jpg]

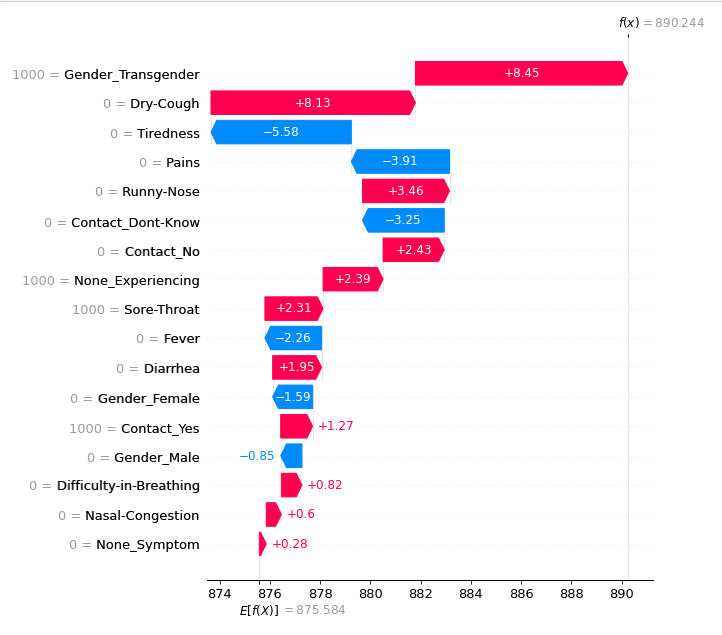

Supplement: Supplementary file 1 [file Data_Sheet_1.ZIP › Fig6b)DT_waterfall.jpg]

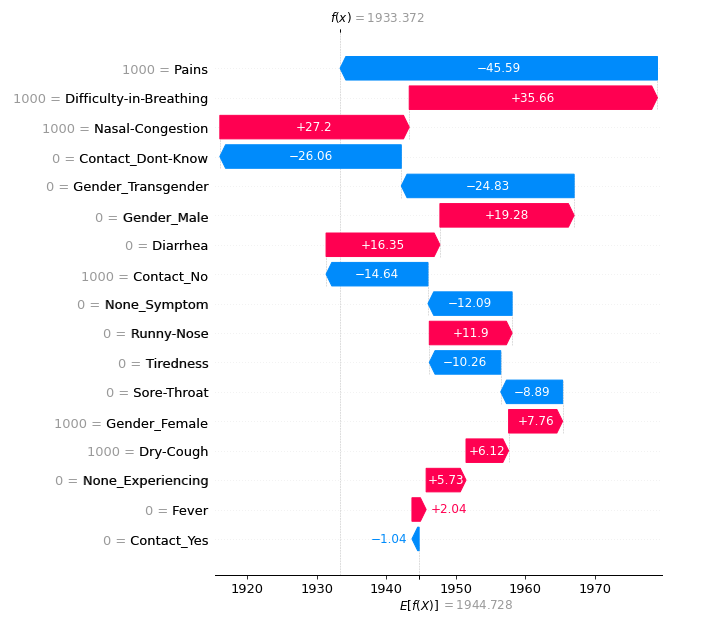

Supplement: Supplementary file 1 [file Data_Sheet_1.ZIP › Fig6a)XG_waterfall.jpg]

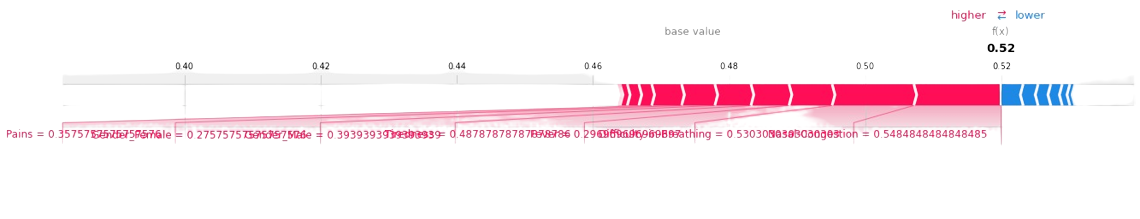

Supplement: Supplementary file 1 [file Data_Sheet_1.ZIP › Fig5c)ANN_fplot.jpg]

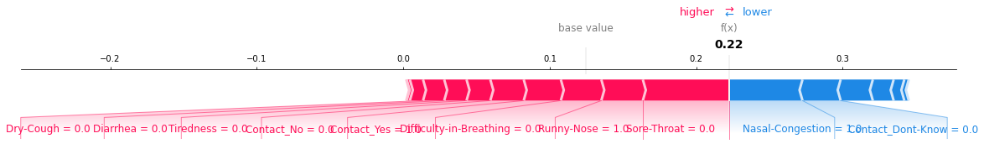

Supplement: Supplementary file 1 [file Data_Sheet_1.ZIP › Fig5b)DT_fplot.jpg]

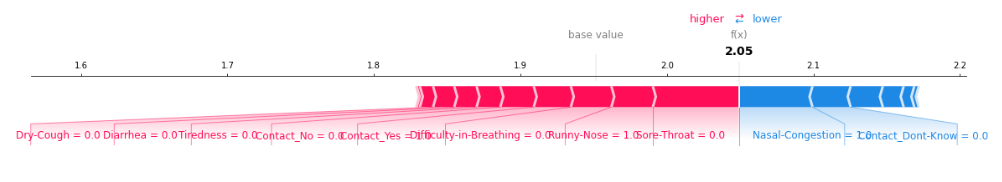

Supplement: Supplementary file 1 [file Data_Sheet_1.ZIP › Fig5a)XG_fplot.jpg]

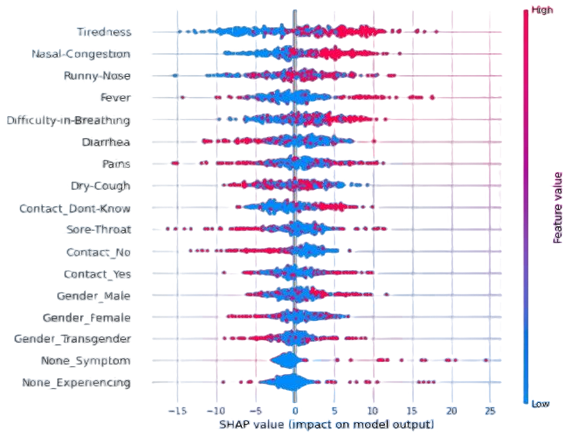

Supplement: Supplementary file 1 [file Data_Sheet_1.ZIP › Fig4c)ANN_beeswarm.jpg]

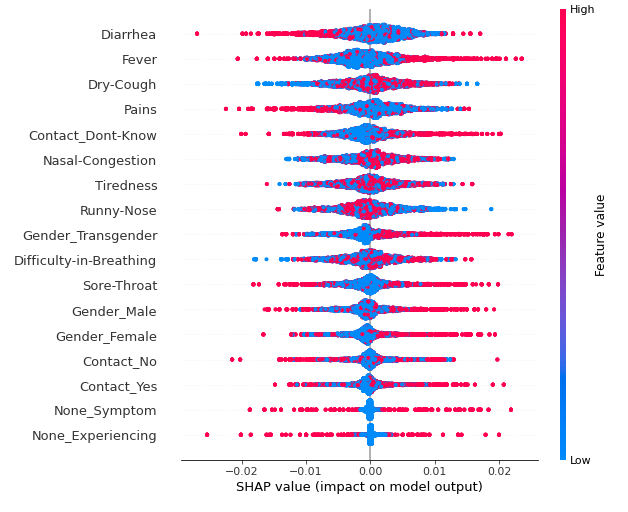

Supplement: Supplementary file 1 [file Data_Sheet_1.ZIP › Fif4b)DT_beeswarm.jpg]

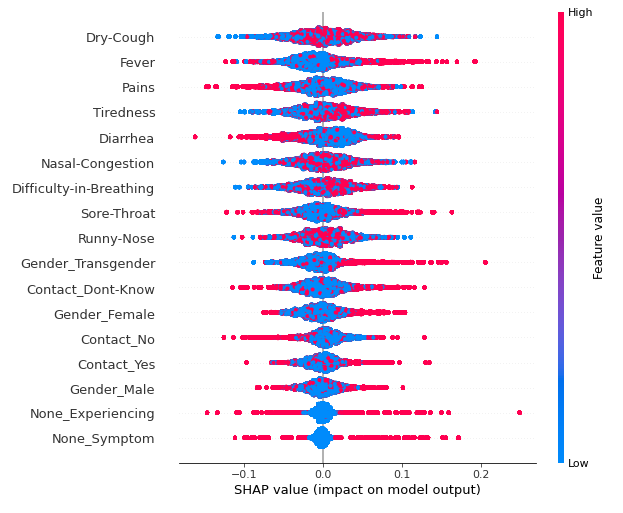

Supplement: Supplementary file 1 [file Data_Sheet_1.ZIP › Fig4a)XG_beeswarm.jpg]

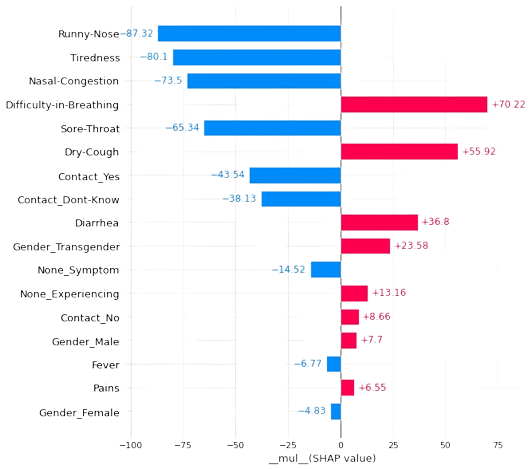

Supplement: Supplementary file 1 [file Data_Sheet_1.ZIP › Fig3c)ANN_lplot.jpg]

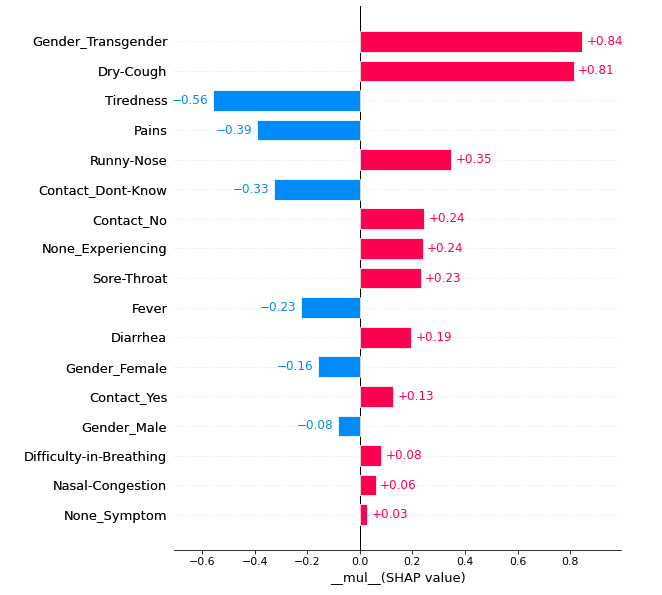

Supplement: Supplementary file 1 [file Data_Sheet_1.ZIP › Fig3b)DT_lplot.jpg]

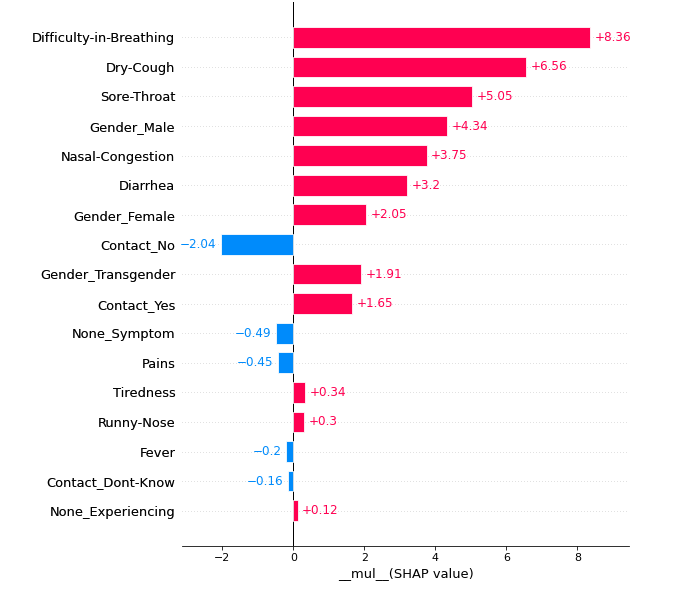

Supplement: Supplementary file 1 [file Data_Sheet_1.ZIP › Fig3a)XG_lplot.jpg]

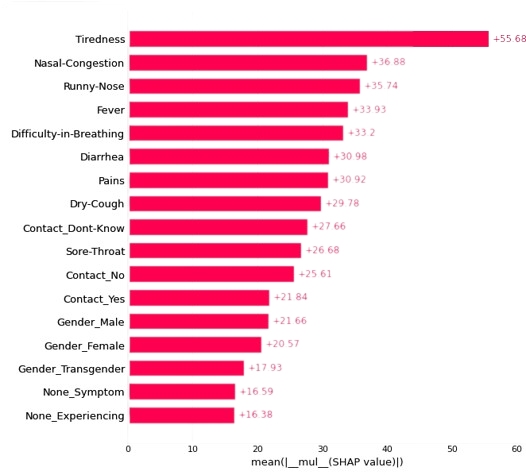

Supplement: Supplementary file 1 [file Data_Sheet_1.ZIP › Fig2c)AN_gbplot.jpg]

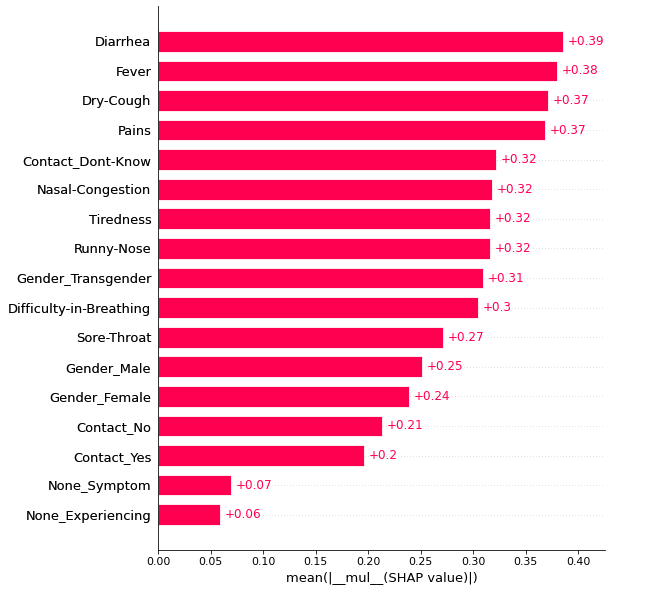

Supplement: Supplementary file 1 [file Data_Sheet_1.ZIP › Fig2b)DT_gplot.jpg]

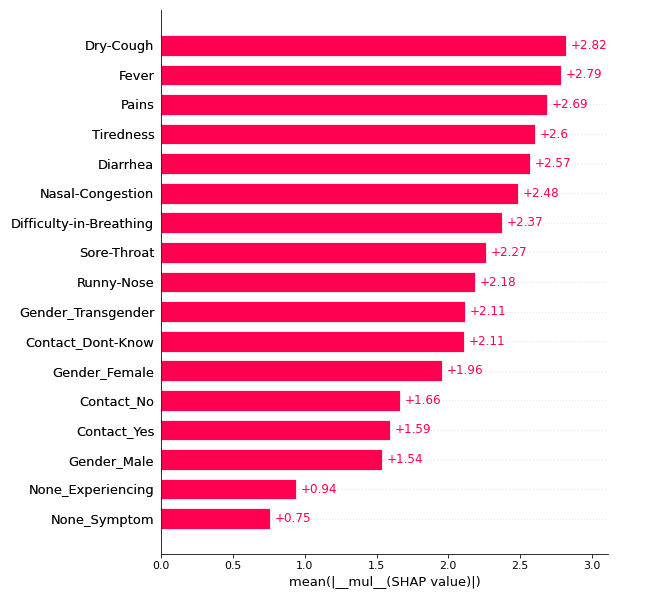

Supplement: Supplementary file 1 [file Data_Sheet_1.ZIP › Fig2a)XG_gplot.jpg]
